# Supplementary material for: Institutional delivery and its determinants among reproductive-age women in Mozambique: A geographic weighted regression
Source: PLOS Glob Public Health. 2025 Aug 28;5(8):e0004520. doi: 10.1371/journal.pgph.0004520 (PMC12393721; doi:10.1371/journal.pgph.0004520)
Supplement: Attachment — Submitted filename: Resonse to Reviewers.docx [file pgph.0004520.s002.docx]

**Response to reviewers and editor comments**

PGPH-D-24-03063

**Title**: Institutional delivery and its determinants among reproductive-age women in Mozambique: A Geographic weighted regression

**Journal**: PLOS Global Public Health

**Authors:** *Alebachew Ferede Zegeye, Habtu Kifle Negash, Alemu Teshale, Mequanint Kassa, Woretaw Endalew, Belete Assefa, Araya Mesfin Nigatu,* *Solomon Gedlu Nigatu*

**Subject:** Submission of revised manuscript

Yours sincerely,

**Alebachew Ferede Zegeye (on behalf of all authors)**

University of Gondar, Gondar, Ethiopia

**Response to reviewers and editor comments**

We are very welcome to accept the revisions requested by the reviewers and editors of this manuscript. Please see below our detailed modifications for the manuscript (PGPH-D-24-03063). Also, kindly find the attached a clean revised manuscript and a revised manuscript with tracked changes.

**Journal Requirements:**

1. We have amended your Competing Interest statement and Funding Disclosure to comply with journal style. We kindly ask that you double check the statement and let us know if anything is incorrect.

- **Response**: Dear Editor, thank you for updating the Competing Interest statement and Funding Disclosure to align with the journal's style. We have carefully reviewed the amendments and confirm that it is accurate. No further changes are required.

2. Please send separate figure files in .tif or .eps format. Also, remove the figures from your manuscript file but keep the legends.

- **Response**: Dear Editor, thank you for your feedback. We have converted all figures into TIF format and submitted them as separate files as per the journal’s requirements. Additionally, we have removed the figures from the main manuscript while retaining the figure legends in the appropriate section.

3. Thank you for uploading your study's underlying data set. Unfortunately, the repository you have noted in your Data Availability statement does not qualify as an acceptable data repository according to PLOS's standards. At this time, please upload the minimal data set necessary to replicate your study's findings to a stable, public repository (such as figshare or Dryad) and provide us with the relevant URLs, DOIs, or accession numbers that may be used to access these data. For a list of recommended repositories and additional information on PLOS standards for data deposition

- **Response**: Dear Editor, thank you for your feedback and for reviewing our manuscript. We acknowledge the journal’s data availability requirements and appreciate the opportunity to ensure compliance. We have uploaded the minimal dataset used for our analysis in Microsoft Excel format.

4. Some material included in your submission may be copyrighted. According to PLOS’s copyright policy, authors who use figures or other material (e.g., graphics, clipart, maps) from another author or copyright holder must demonstrate or obtain permission to publish this material under the Creative Commons Attribution 4.0 International (CC BY 4.0) License used by PLOS journals. Please closely review the details of PLOS’s copyright requirements here: PLOS Licenses and Copyright. If you need to request permissions from a copyright holder, you may use PLOS's Copyright Content Permission form.

- Please respond directly to this email or email the journal office and provide any known details concerning your material's license terms and permissions required for reuse, even if you have not yet obtained copyright permissions or are unsure of your material's copyright compatibility.

Potential Copyright Issues:

- “Figures 2, 3, 4, 5, 6, 7, 8”: please (a) provide a direct link to the base layer of the map (i.e., the country or region border shape) and ensure this is also included in the figure legend; and (b) provide a link to the terms of use / license information for the base layer image or shapefile. We cannot publish proprietary or copyrighted maps (e.g. Google Maps, Mapquest) and the terms of use for your map base layer must be compatible with our CC-BY 4.0 license.
- Note: if you created the map in a software program like R or ArcGIS, please locate and indicate the source of the basemap shapefile onto which data has been plotted.
- If your map was obtained from a copyrighted source please amend the figure so that the base map used is from an openly available source. Alternatively, please provide explicit written permission from the copyright holder granting you the right to publish the material under our CC-BY 4.0 license.
- Please note that the following CC BY licenses are compatible with PLOS license: CC BY 4.0, CC BY 2.0 and CC BY 3.0, meanwhile such licenses as CC BY-ND 3.0 and others are not compatible due to additional restrictions.
- **Response**: Dear Editor, thank you for your detailed review and for bringing the copyright concerns to our attention. We have carefully reviewed the materials used in our submission and would like to provide the following clarifications regarding the base layers of the maps in Figures 2, 3, 4, 5, 6, 7, and 8.
- **Source of the base layer:**
- The base layer of the maps was obtained from the Humanitarian Data Exchange (HDX): <https://data.humdata.org/dataset/5e8d83a5-1210-49be-b7d9-cf286dbc15df>.
- This source provides openly available spatial data and is included in the figure legends as required.
- **License compliance:**
- The dataset is available under an open and permissible license that allows reuse, and we have ensured compliance with PLOS’s CC-BY 4.0 licensing requirements.
- **Map creation and processing:**
- The maps were generated using ArcGIS with the referenced shapefiles.
- We confirm that no proprietary or copyrighted maps (e.g., Google Maps, MapQuest) were used in the manuscript.

**Reviewer 1 Comments:**

1. Line 115-119: Sampling process to explicitly mention how a figure of 619 enumeration areas was decided. What was the sampling frame and the sampling interval for the selection of households? Were any inclusion and exclusion criteria used for selecting women from the households? Total number of household / women whose data were analyzed must be mentioned in the methodology.

- **Response**: Dear reviewer, First and foremost, thank you for your in-depth review of our manuscript and for your insightful comments. We have explicitly modified the sampling process in the revised manuscript. Specifically, we have detailed how the 619 enumeration areas were determined, the sampling frame, and the selection criteria for households and women. Additionally, we have included the total number of households and women whose data were analyzed. Kindly see the amendments on page 5, lines 115-123 in the revised manuscript.

2. Line 179: Operational definitions of primary and secondary clusters to be mentioned in the methodology.

- **Response**: Dear Reviewer, thank you for your recommendations. We have revised the methodology section to define primary and secondary clusters. Kindly see the amendments on page 7, lines 182–185.

3. Line 204: What was the reason behind selecting the cut-off of 7.5 for VIF? Generally accepted values are either 5 or 10. Kindly explain and include it in the manuscript.

- **Response**: Dear Reviewer, thank you for bringing to our attention. The VIF cut-off value of 7.5 was specifically chosen based on the context of Ordinary Least Squares (OLS) regression, where some studies have suggested that a more conservative threshold, such as 7.5, may be appropriate to detect multicollinearity while balancing the risk of excluding potentially meaningful variables. We have cited relevant references that support this evidence, which indicate that the threshold of 7.5 can be more suitable for OLS regression models. Please see the explanations on page 8, line 211-114 of the revised manuscript.

4. Line 209: Full form of GWR to be mentioned first and then the abbreviation to be mentioned in the parenthesis.

- **Response**: Dear reviewer, Thank you for your comment. We have revised the sentence to include the full form of GWR before the abbreviation. Kindly see the corrections in the revised manuscript on page 8, lines 118-121.

5. Line 221: Avoid using terms like ‘big problem’ in the results unless some objective criteria were used for quantification of the problem. Any cut off distance was used for this classification? If not, rephrase the sentence appropriately.

- **Response**: Dear Reviewer, Thank you for your thoughtful feedback. We acknowledge that the term "big problem" is subjective and have revised the sentence to ensure clarity and objectivity. Since no specific cut-off distance was used for classification, we have rephrased the text on page 9, lines 231-232 of the revised manuscript.

6. Line 224: Table 1 – Sex of the household head- ‘meal’ to be replaced with “male”.

- **Response**: Dear Reviewer, thank you for pointing out this typographical error. We have corrected “meal” to “male” in Table 1. We appreciate your careful attention to detail. Kindly see the correction in Table 1 of the revised manuscript.

7. Line 250-251: High prevalence was reported in northwestern part of the country (as evidenced from the map: refer to Niassa province) and not northeastern as mentioned in the text! Check and modify accordingly.

- **Response**: Dear reviewer, Thank you for your careful review and for pointing out this point. After carefully reviewing the map, we have corrected the text accordingly. Kindly see the amendments on page 11, lines 259-264 of the revised manuscript.

8. Line 273: Is the last word of this line ‘high’ required? If not, omit.

- **Response**: Dear Reviewer, Thank you for your insightful comment. As per your suggestion, we have removed the word “high” from the sentence. Kindly see the revised version of the manuscript on page 14, line 295.

9. Line 279: Replace the word ‘vary’ with ‘varying’

- **Response**: Dear Reviewer, Thank you for your helpful suggestion. We have revised the text by replacing the word "vary" with "varying" on page 15 line 300 as recommended.

10. Line 291: Table 3 – For the ease of comprehension it would be better to include only the value of the coefficient and the p value for the variables in the table. The other statistics may be omitted.

- **Response**: Dear Reviewer, Thank you for your insightful comment. We understand the suggestion to simplify Table 3 by including only the coefficient values and p-values for ease of comprehension. However, based on common practice in literature for spatial analysis using Geographic Weighted Regression (GWR), we have chosen to retain additional statistics such as Standard Error, t-statistic, Probability, Robust SE, Robust t-statistics, robust probability, and VIF values. These statistics are commonly included in GWR analyses for comparison and to provide a more comprehensive view of the model's robustness and the relationship between variables.

We agree that clarity is important, and therefore, we have revised Table 3 to ensure that the essential statistics are displayed clearly and concisely, while also keeping the additional statistics for comparison purposes. We believe this approach adds value to the interpretation of the GWR results. However, if the reviewer still feels that the table is overly complex, we are open to further adjustments to better meet the needs of the readers.

11. Line 296: ‘R2’ to be replaced with R square.

- **Response**: Dear Reviewer, Thank you for your helpful suggestion. We have replaced ‘R2’ with the more formal term "R square" as recommended. Kindly see the amendments on page 16, line 317-318 in the revised manuscript.

12. Line 305 – 306: Check the sentence – As shown in Figure 7 …it appears incoherent – modify appropriately.

- **Response**: Dear Reviewer, we appreciate your careful attention to clarity. In response to the comment, we have revised the sentence for coherence and precision. Kindly see the amendments on page 16, lines 327-329.

13. Line 307: Replace the word ‘whose’ with word ‘with’.

- **Response**: Dear Reviewer, we appreciate your careful attention to detail. As suggested, we have replaced the word ‘whose’ with ‘with’ on page 16 Line 329 of the revised manuscript.

14. . Line 446: References poorly compiled, use standard Vancouver style

- Ref 1: Replace Organization WH with World Health Organization.
- Ref 3: replace word ‘retrieved’ with ‘cited on’.
- Ref 4: check page number; it can’t be 1 to 0, if it is 1 to 10, it must be written as such.
- Ref 5: Rewrite ‘Bjog’ as per the standard format.
- **Response**: Dear Reviewer, we thank you for your careful attention to the reference formatting. All requested updates have been implemented in strict accordance with Vancouver style guidelines. Specifically, in Reference 1, "Organization WH" was replaced with the full name "World Health Organization." In Reference 3, the term "retrieved" was revised to "cited on" for accuracy. For Reference 4, the erroneous page range "1–0" was corrected to "1–10." Lastly, in Reference 5, the journal title "Bjog" was reformatted to the standard abbreviation (BJOG). We have further verified the entire reference list to ensure uniformity and adherence to Vancouver standards. Kindly see the amendments in the revised manuscript.

**Reviewer 2 Comments:**

Major Comments:

1. The paper talks about different factors affecting hospital births but should explain more about how spatial variability influence these factors.

- **Response**: Dear reviewer, First and foremost, we would like to express our sincere gratitude for your insightful comments and suggestions, which have significantly contributed to the improvement of this manuscript. In response to your suggestions, we have expanded our discussion to explicitly highlight the geographic patterns observed in our analysis. The geographically weighted regression (GWR) results revealed significant spatial variation in how key factors like wealth, transportation, and skilled birth attendance influence institutional delivery rates. For instance, the positive association between wealth and institutional delivery was strongest in remote regions like Cabo Delgado and Niassa, where economic disparities create sharper divides in healthcare access. Similarly, vehicle transportation emerged as particularly crucial in areas with poor infrastructure, such as Tete, while its impact was less pronounced in urban centers like Maputo where facilities are more accessible. The availability of skilled birth attendants showed the greatest effect in rural "cold spot" regions where workforce shortages are most acute.

2. The methods section is clear, but it needs to explain:

a. How spatial weights are chosen in geographically weighted regression.

b. Why Kriging interpolation was used over other available methods?

c. If a sensitivity analysis done to check the accuracy of the results?

- **Response**: Dear Reviewer, thank you for your attention to detail. To clarify, in the geographically weighted regression (GWR), spatial weights were determined using an adaptive kernel bandwidth, optimized via the corrected Akaike Information Criterion (AICc) to account for clustered observations. Kriging interpolation was selected for its demonstrated accuracy in spatial prediction, as reflected in its lower residuals and root mean square error compared to alternative methods. While sensitivity analysis was not performed for temporal variations or other reasons (as data were collected during the same period), the robustness of our spatial models was ensured through rigorous validation, including comparison of GWR with global OLS results and significance testing of spatial clusters via Monte Carlo simulations in SatScan. Kindly see in lines 224-247, 178-181 respectively.

3. The paper uses good spatial analysis, but it should clarify:

a. Why an adaptive kernel was used for GWR.

b. How the model handles the issue of locations being too similar to each other.

c. The importance of clusters in the Sat Scan study.

- **Response**: Dear Reviewer, thank you for your suggestions. (a) An adaptive kernel was selected for GWR due to the clustered distribution of observations, ensuring optimal bandwidth adjustment to local density variations and improving model accuracy. (b) To address potential over-similarity of nearby locations, we validated spatial independence using Moran’s I and employed robust standard errors in GWR to mitigate autocorrelation effects. (c) The SatScan clusters identified significant spatial patterns of institutional delivery, highlighting priority regions (example, Maputo, Sofala) where targeted interventions could enhance service utilization. Therefore, these clusters underscore the geographical heterogeneity of healthcare access, aligning with the study’s goal of informing spatially custom-made policies. Kindly see the amendments on page 9, lines 226-229 of the revised manuscript.

4. The discussion compares findings with other studies but should explain more about why Mozambique exhibits such spatial variations.

- **Response**: Dear Reviewer, thank you for bringing this to our attention. We have expanded the discussion to provide a more detailed explanation of why Mozambique exhibits significant spatial variations in institutional delivery. Kindly see the revised manuscript on page 18, line 376-382.

5. The conclusion should include suggestions on improving hospital birth rates.

- **Response**: Dear Reviewer, we thank you for your recommendations. As suggested, we have revised the Conclusion section to include specific recommendations. Kindly see the amendments on page 20, lines 440-443 in the revised manuscript.

**Minor Comments: Rephrase the following sentences for clarity**

1. Line 56: "Utilizing services provided …. health of mothers" … for example … Utilizing medical institutions for childbirth is a crucial strategy to enhance maternal health.

- **Response**: Dear Reviewer, we appreciate your constructive suggestion to improve clarity. As recommended, we have rephrased the sentence on page 3, Line 56-57 in the revised manuscript.

2. Line 63: "The World Health Organization identifies … causes of these fatalities".

- **Response**: Dear reviewer, Thank you for your insightful comments and suggestions regarding our manuscript. As suggested, we have rephrased the sentence for greater clarity. Kindly see the amendments on page 3, lines 60-63 of the revised manuscript.

3. Line 78: "On a worldwide scale, around 830 … pregnancy and childbirth."

- **Response**: Dear reviewer, thank you for your attention to detail in reviewing our manuscript. We have taken your suggestion into account and have made the correction. Kindly see the amendments on page 3, lines 76-77 of the revised manuscript.

4. Line 110: "The study in question utilized … Mozambique Demographic and Health Survey.".

- **Response**: Dear reviewer, thank you for your comment. Please see the amendment on page 5, lines 110-111 of the revised manuscript.

5. Line 123: "Mozambique’s administrative structure … provincial status" … for example, Mozambique consists of ten provinces and Maputo City, which has provincial status.

- **Response**: Dear reviewer, thank you for your observation. In the revised manuscript, we have rephrased the section. Kindly see the amendment on page 5, line 126.

6. Line 133: "This variable was categorized into two: ‘home delivery’, which includes births took place at the respondent’s own home or another home, and ‘institutional delivery’..." → replace “took place” with “that occurred”

- **Response**: Dear reviewer, thank you for directing us to rephrasing the section. Please see the amendments on page 5, lines 134-137.

7. Line 147: "Data cleaning and preparation were conducted using STATA version 17 and Microsoft Excel … replace “conducted' with “performed”

- **Response**: Dear reviewer, Thank you for your careful reading and for pointing out this typographical error. We have revised the manuscript to reflect this correction. Please see the correction on page 6, line 151.

8. Line 162: "A Moran’s I value close to -1 … no particular pattern." → A Moran’s I value near -1 suggests a dispersed distribution of institutional deliveries, indicating no specific pattern.

- **Response**: Dear reviewer, we thank for this helpful suggestion to improve clarity. As requested, we have rephrased the sentence for better precision. Kindly see the correction on page 6, lines 165-167.

9. Line 222: "Less than half (46.76%) of women had a big problem to access healthcare institutions due to distance" → Less than half (46.76%) of women faced significant difficulties accessing healthcare institutions due to distance.

- **Response**: Dear reviewer, thank you for your suggestion to improve clarity in the sentence. As recommended, we have rephrased the text for better readability and precision. Kindly see the corrections on page 9, lines 131-132 of the revised manuscript.

10. Line 252: "In the Kriging interpolation,… southern parts of Mozambique." → Kriging interpolation identified a high predicted prevalence of institutional delivery in the northeastern, central, and southern regions of Mozambique.

- **Response**: Dear reviewer, thank you for your attention to this manuscript accuracy. Kindly see corrected section on page 11, line 259.

11. Line 331: "Despite global efforts, low utilization … and cultural factors." → Despite global efforts, institutional delivery services remain underutilized in many low- and middle-income countries due to socio-demographic, economic, and cultural factors.

- **Response**: Dear Reviewer, thank you for your valuable suggestion to improve clarity. We have revised the sentence in on page 17, lines 349-350.

12. Line 411: "Accordingly, hot spot regions for institutional delivery were detected in Maputo city, southwest Inhambane, central Sofala, southern Gaza, and western Niassa region." → Hotspot regions for institutional delivery were identified in Maputo City, southwest Inhambane, central Sofala, southern Gaza, and western Niassa.

- **Response**: Dear Reviewer, we thank you for this helpful suggestion to improve clarity. As recommended, we have rephrased the sentence on page 20 lines 434-436.

13. Line 415: "Spatial regression analysis … emerging regions of Mozambique." → Spatial regression analysis identified wealth index, unwanted pregnancy, vehicle transportation, and the presence of skilled birth attendants as significant determinants of institutional delivery in Mozambique.”

- **Response**: Dear Reviewer, thank you for your suggestion to enhance clarity in this sentence. We have revised the section. This change has been made on page 20, line 436-438.

14. The study is methodologically sound and makes an important contribution to understanding institutional delivery in Mozambique. Clarifications in methodology, improved discussion structure, and a stronger conclusion would all considerably increase the manuscript's quality.

- **Response**: Dear reviewer; we sincerely thank you for your positive feedback and constructive suggestions. We have tried to carefully address all points by clarifying the methodology, restructuring the discussion, and strengthening the conclusion with actionable recommendations. Thank you!
